# From brain to heart: cognitive performance shapes exercise- induced cardiac autonomic trajectories in older adults

**DOI:** 10.3389/fmed.2026.1761743

**Published:** 2026-01-28

**Authors:** Paulina Sepúlveda-Figueroa, Matías Castillo-Aguilar, Alexis Sepúlveda-Lara, Cristian Sandoval, Constanza Saavedra, Cristian Núñez-Espinosa

**Affiliations:** 1 Departamento de Ciencias Preclínicas, Facultad de Medicina, Universidad de La Frontera, Temuco, Chile; 2 Centro Asistencial Docente e Investigación (CADI-UMAG), Universidad de Magallanes (UMAG), Punta Arenas, Chile; 3 Escuela de Medicina, Universidad de Magallanes (UMAG), Punta Arenas, Chile; 4 Doctorado en Ciencias Mención Biología Celular y Molecular Aplicada, Facultad de Ciencias Agropecuarias, Universidad de La Frontera, Temuco, Chile; 5 Escuela de Tecnología Médica, Facultad de Salud, Universidad Santo Tomás, Los Carreras, Osorno, Chile; 6 Departamento de Medicina Interna, Facultad de Medicina, Universidad de La Frontera, Temuco, Chile; 7 Servicio de Neurología, Hospital Clínico de Magallanes, Punta Arenas, Chile

**Keywords:** aging, autonomic nervous system, body mass index, cognition, heart rate variability

## Abstract

**Background:**

Autonomic dysregulation and cognitive decline often co-occur in aging, but most work uses only resting heart rate variability (HRV). Therefore, we examined whether global cognitive performance modulates dynamic HRV responses to a functional exercise test in community-dwelling older adults.

**Methods:**

In this cross-sectional study, 104 adults aged 60–85 years from southern Chile completed a rest–exercise–recovery protocol using the 2-Min Step Test. Global cognition was assessed with the Addenbrooke’s Cognitive Examination. Short-term HRV (time and frequency domains), parasympathetic (PNS) and sympathetic (SNS) indices, and Baevsky’s Stress Index were derived from Polar H10 recordings at rest, during exercise, and recovery. Hierarchical Bayesian regression models characterized HRV trajectories as a function of cognitive performance, adjusting for body mass index.

**Results:**

Higher cognitive scores were associated with a more favorable autonomic profile at rest, including higher standard deviation of normal-to-normal intervals, low frequency (LF), and very low frequency (VLF) and lower SNS and Stress Index values. During exercise, better cognition was linked to greater high frequency power and larger reductions in LF and VLF from rest to effort, indicating enhanced autonomic flexibility. Lower cognitive scores showed flatter LF/VLF trajectories and a sharper in-exercise decline in the PNS index. Higher body mass index was consistently related to reduced vagal modulation and higher sympathetic markers.

**Conclusion:**

Older adults with better cognitive performance show healthier resting autonomic profiles and greater adaptability of cardiac autonomic regulation during a brief functional test. Blunted HRV trajectories in individuals with lower cognition may signal early neurophysiological vulnerability, and 2-Min Step Test-derived HRV responses may provide a non-invasive marker of cognitive health in aging.

## 1 Introduction

Aging is accompanied by progressive physiological changes, including declines in cognitive performance and alterations in autonomic regulation, typically expressed as reduced vagal activity and increased sympathetic drive (1, 2). Heart rate variability (HRV) is a commonly utilized, non-invasive biomarker that indicates the capacity of the autonomic nervous system (ANS) to regulate cardiac rhythm in response to both internal and external stimuli (3, 4). Lower HRV has consistently been associated with heightened physiological stress, reduced adaptability, and increased morbidity and mortality in older adults (5).

Cognitive function and autonomic cardiovascular control are linked via the central autonomic network (CAN), a dispersed system that includes the prefrontal cortex, insula, anterior cingulate cortex, amygdala, hypothalamus, and brainstem nuclei (6, 7). This network facilitates bidirectional communication between the brain and the circulatory system, allowing higher-order cortical areas to modulate autonomic output, while interoceptive and baroreceptor inputs affect cognitive and emotional functions (8). Malfunction within this network has been identified as a primary mechanism connecting cognitive deterioration with diminished autonomic flexibility (9–11).

A growing body of research suggests that lower resting heart rate variability (HRV) is associated with a higher risk of dementia and moderate cognitive impairment (MCI), as well as decreased attention, working memory, and executive function (12–14). However, most studies only use resting-state HRV, which offers little information about the ANS’s dynamic flexibility. Because physical activity causes the parasympathetic system to shut down, the sympathetic system to turn on, and then the vagal system to turn back on during the recovery phase, heart rate variability responses to exercise and recovery give us more information about how well the autonomic system works (15–17).

Individuals with superior executive or memory function often exhibit enhanced autonomic flexibility, characterized by improved vagal control and expedited recovery from physiological or cognitive stressors (14). Neuroimaging studies bolster the concept of neurovisceral integration by demonstrating shared cortical substrates that link autonomic regulation with cognitive control (18). Moreover, oxidative stress, endothelial dysfunction, and neuroinflammatory pathways may influence cognitive and autonomic processes through age-related structural deterioration, chronic inflammation, and metabolic imbalance (10, 19).

This study aimed to examine the influence of cognitive ability in older adults on their dynamic cardiac autonomic response to exercise. The Addenbrooke’s Cognitive Examination III (ACE-III) was utilized to evaluate cognitive ability, emphasizing language, verbal fluency, orientation, memory, and visuospatial skills. A rest-exercise-recovery regimen was employed to document rapid physiological changes, and multivariate hierarchical Bayesian regression was utilized to analyze HRV trajectories. This study employs a Bayesian modeling approach to extend beyond static HRV indicators, offering a thorough characterization of autonomic trajectories and their relationship with cognitive integrity.

## 2 Materials and methods

### 2.1 Study design

This study employed a multifaceted design that examined various perspectives, identified patterns, and included a diverse population sample. Prior to the commencement of data collection, all participants received a comprehensive overview of the study’s objectives, methodologies, and potential risks and benefits. Informed consent was obtained from each participant in accordance with ethical guidelines to protect individual autonomy. The protocol followed the principles established in the Declaration of Helsinki, and ethical approval was obtained from the institutional ethics committee.

Data collection occurred at two academic healthcare and research institutions: (i) the Centro Asistencial Docente e Investigación (CADI-UMAG), affiliated with the Universidad de Magallanes in Punta Arenas, Chile, and (ii) Universidad de La Frontera in Temuco, Chile. Assessments were conducted from 9:00 to 11:00 a.m. to minimize potential circadian variations in physiological or cognitive outcomes (20).

Testing occurred in controlled environments to maintain methodological consistency. The evaluation room was kept at a constant temperature of 20°C, and artificial white lighting was employed to standardize illumination, thereby minimizing variability in ambient conditions that could affect visual or cognitive performance. All procedures were conducted in a private, quiet environment to reduce external distractions and improve measurement reliability.

### 2.2 Participants

Participants were recruited using a non-probabilistic convenience sampling method through community advertisements and outreach initiatives. The last group of people included 104 older adults, 84 of whom were women and 20 of whom were men. Eligibility criteria included: (i) Participants must be 60 years of age or older at enrollment; (ii) must have permanent residence in either the Magallanes and Chilean Antarctic Region or the La Araucanía Region (Temuco), ensuring comparable environmental and socioeconomic contexts; (iii) must achieve a score > 60% on the Karnofsky Performance Status scale, indicating sufficient functional capacity and independence to complete all study procedures (21); and (iv) must not have medical conditions that could interfere with cardiovascular or autonomic function, including diabetic neuropathy, pacemaker implantation, clinical depression, cognitive impairment, motor disability, or dementia.

### 2.3 Measurement procedure

Participants underwent a single evaluation session following a 12-h overnight fast, during which they abstained from strenuous physical activity and alcohol consumption. Upon arrival, written informed consent was confirmed, and a brief health screening was conducted, which included the verification of eligibility criteria and an assessment of resting blood pressure (systolic < 130 mmHg; diastolic < 80 mmHg). Data on sociodemographic factors and medical history were obtained via a structured interview. Body composition was initially evaluated utilizing a Tanita BC-558 multi-frequency bioelectrical impedance analyzer (BIA). Measurements adhered to manufacturer protocols, necessitating those participants fast for a minimum of 4 h, void their bladder, and stand barefoot while grasping the hand electrodes.

The Two-Minute Step Test (TMST) was administered by a qualified kinesiologist after the subject had been still for at least 10 min. To ensure the participants’ safety, blood pressure was immediately measured again following the TMST. Cognitive function was then assessed in a quiet, controlled environment using the Spanish-validated version of the ACE-III. The ACE-III was administered by a skilled assessor who had received training in standardized cognitive evaluation methods. Every tool was utilized in accordance with stated and agreed norms.

### 2.4 Assessments

#### 2.4.1 Body composition

Body composition was assessed utilizing multi-frequency BIA equipment. The estimates provided include BMI, fat mass percentage, lean mass, total body water, and bone mass. Measurements were obtained using the Tanita BC-558 Ironman Segmental Body Composition Monitor (Tanita Corporation, Tokyo, Japan).

Before BIA testing, participants were instructed to fast for a minimum of 4 h and to refrain from strenuous exercise and fluid consumption for at least 12 h. Prior to measurement, participants were instructed to void their bladders. Participants donned light clothing and contacted the foot electrodes of the device while barefoot during the test. The hand electrodes were also held by them.

#### 2.4.2 Addenbrooke’s cognitive examination

The ACE-III was utilized to assess overall cognitive function. This neurocognitive test is frequently employed in clinical and research contexts to identify MCI and dementia (22). The tool has undergone translation, cultural adaptation, and psychometric validation in multiple languages, including Spanish (23, 24).

Based on five domains as attention and orientation, memory, verbal fluency, language, and visuospatial abilities; the ACE-III is a cognitive screening tool that yields a total score of 100 points. Higher scores indicate improved cognitive function.

Trained evaluators administered the Spanish version following standardized procedures. When clarification was required, instructions were delivered using simple and culturally appropriate language while maintaining the original format and scoring criteria. All participants completed the full assessment in a single session within a quiet, controlled environment. The total ACE-III score served as the primary indicator of global cognitive functioning.

Interpretation of ACE-III scores followed the Argentinian–Chilean validation study by Bruno et al., which recommended a cut-off score of 86/100 for identifying dementia in this population. Using this threshold, the instrument correctly classified 98.6% of Alzheimer’s disease cases, 83.9% of behavioral-variant frontotemporal dementia cases, and 84.2% of healthy controls (24).

#### 2.4.3 Two-minute step test

To elicit an acute, submaximal physiological challenge and assess functional capacity, the TMST was employed. This test is a standardized component of the Senior Fitness Test battery developed by Rikli and Jones (25). In the present study, the TMST functioned as a controlled exercise stimulus to examine dynamic cardiac autonomic responses in older adults while simultaneously providing an index of lower-limb endurance and functional mobility.

Participants were instructed to march in place for 2 min, raising each knee to a height corresponding to the midpoint between the patella and the iliac crest. A trained kinesiologist supervised the procedure, provided standardized verbal cues, and ensured correct stepping technique. The total number of steps completed during the 2-min interval was recorded as the functional performance measure. Short pauses were permitted if necessary, and participants were encouraged to resume the movement as soon as they were able.

Continuous R–R interval monitoring was performed throughout the TMST using Polar H10 heart rate sensors (Polar H10; Polar Electro Oy, Kempele, Finland) to characterize HRV before, during, and after the exercise bout. Blood pressure was assessed immediately before and immediately after the test with an automated monitor (Omron HEM-7142; Omron Healthcare Co., Ltd., Kyoto, Japan) to ensure safety and document acute hemodynamic changes.

The TMST was administered following a minimum 10-min seated or standing rest period and was followed by a 5-min passive recovery interval (total protocol duration: approximately 12 min). The number of steps completed constituted the primary functional capacity outcome, whereas the physiological disturbance induced by the TMST served as the basis for evaluating dynamic autonomic regulation.

#### 2.4.4 Cardiovascular parameters

HRV was calculated from R–R interval recordings obtained using the Polar Team2 system (Polar Electro Oy, Kempele, Finland). Participants rested in a supine position, and continuous R–R data were collected during the final 10 min of the resting period. From these recordings, a 5-min artifact-free segment was selected for analysis in accordance with established recommendations (11, 26).

Data processing was performed with Kubios HRV^®^ software (version 3.5.0; Kubios Oy, Kuopio, Finland). Artifact correction was applied using the “Low” automatic threshold with cubic spline interpolation. Segments in which more than 5% of beats required correction were excluded; those with ≤ 5% corrected beats were retained, with the correction rate documented. Slow non-stationary trends were removed using the Kubios smoothness priors detrending approach (third-order polynomial; default λ for 5-min segments).

Time-domain metrics included the root mean square of successive differences between adjacent R–R intervals (RMSSD, ms), an index of parasympathetic (vagal) modulation (27), and the standard deviation of normal-to-normal intervals (SDNN, ms), a measure reflecting global HRV influenced by both sympathetic and parasympathetic inputs (28, 29). The Parasympathetic Nervous System (PNS) index, Sympathetic Nervous System (SNS) index, and Baevsky’s Stress Index (SI) were additionally computed using the algorithms provided in Kubios. The PNS index is derived from mean R–R, RMSSD, and the Poincaré plot short-axis standard deviation (SD1), whereas the SNS index incorporates mean R–R, SI, and the long-axis standard deviation (SD2). Both indices are expressed as normalized deviations relative to reference population values (30–32).

For frequency-domain analysis, power spectral density was estimated using Welch’s periodogram with a Hanning window, 50% overlap, and resampling at 4 Hz. Spectral power was computed within the standard frequency bands: very low frequency (VLF, 0.0033–0.04 Hz), low frequency (LF, 0.04–0.15 Hz), and high frequency (HF, 0.15–0.40 Hz). Absolute power values (ms^2^) and, where appropriate, their logarithmic transformations are reported.

Respiration was spontaneous; therefore, breathing rate and/or the HF peak frequency were incorporated as covariates. To ensure physiological validity, the researchers verified that the HF peak remained within the 0.15–0.40 Hz range. Sensitivity analyses were subsequently conducted, excluding any observations in which the HF peak fell outside this interval.

### 2.5 Statistical analysis

#### 2.5.1 Framework

A fully Bayesian modeling framework was used to examine how cardiac autonomic modulation responds to exercise while accounting for the influence of multiple potential confounders. A Bayesian approach was selected instead of traditional frequentist methods because it yields full posterior distributions for all parameters, enabling comprehensive characterization of uncertainty and probabilistic interpretation through credible intervals (33).

For descriptive analyses, continuous variables are presented as means and standard deviations (M ± SD), whereas categorical variables are summarized using absolute frequencies (n) and percentages (%). Initial associations, unadjusted for confounding factors, were evaluated using Spearman’s rho (ρ) with corresponding two-sided *p*-values.

The prior distributions for the linear coefficients were specified as weakly regularizing normal distributions centered at zero [β ∼ *N*(0, 3)]. These priors served to constrain implausible parameter estimates, reduce sensitivity to extreme values, and facilitate more stable convergence during model fitting.

#### 2.5.2 Parameter estimation

Bayesian estimation was conducted using the No-U-Turn Sampler (NUTS), a Hamiltonian Monte Carlo variant, as implemented in the *brms* (v2.22.0) and *rstan* (v2.32.7) packages within R (v4.5.0; R Foundation for Statistical Computing, Vienna, Austria). For each multivariate model, four Markov chains were executed, consisting of 10,000 warm-up iterations followed by 10,000 sampling iterations. To reduce autocorrelation among successive draws, every eighth iteration was retained, yielding 5,000 post–warm-up samples per parameter.

Convergence and sampling stability were evaluated using multiple diagnostic criteria. All parameters were required to have a potential scale reduction factor ($\hat{\text{R}}$) below 1.01 and an effective sample size > 1,000. Trace plots were visually examined to verify adequate mixing across chains, and posterior predictive checks were performed to assess the agreement between model-generated predictions and the observed data.

#### 2.5.3 Statistical reporting

Inference was conducted using the SEXIT (Sequential Effect eXistence and Significance Testing) framework (34). For each parameter and its posterior distribution, the posterior median and the corresponding 95% highest-density credible interval (HDI) are reported. The probability of direction (pd) is provided as a quantitative indicator of effect existence. Practical significance (ps) is expressed as the proportion of the posterior distribution falling outside the region of practical equivalence (ROPE). The ROPE was defined as ± 0.1 times the standard deviation of the response variable, and all predictors were standardized prior to model estimation to ensure comparability and interpretive consistency (34).

## 3 Results

### 3.1 Sample characteristics

The final sample comprised 104 older adults, including 20 men and 84 women. Participants ranged in age from 60 to 85 years, with a mean age of 69.5 ± 6.0 years. Additional demographic and clinical characteristics, stratified by sex, are presented in Table 1.

**TABLE 1:** Basic demographic characteristics and cognitive functioning levels of the collected sample.

Characteristic	Overall	Women	Men	Difference^2^	95% CI^2^
	*n* = 104^1^	*n* = 84^1^	*n* = 20^1^		
Age (years)	69.45 ± 5.96	69.17 ± 6.12	70.65 ± 5.17	−0.27	−0.75, 0.22
Weight (kg)	72.23 ± 13.24	69.07 ± 11.10	85.35 ± 13.60	−1.3	−1.9, −0.81
Height (cm)	159.88 ± 8.25	157.53 ± 6.34	170.16 ± 7.88	−1.8	−2.4, −1.2
BMI (kg/m^2^)	28.17 ± 4.56	27.87 ± 4.51	29.46 ± 4.70	−0.35	−0.85, 0.15
Systolic blood pressure (mm/hg)	131.52 ± 15.54	128.94 ± 15.33	142.00 ± 11.77	−0.97	−1.5, −0.46
Diastolic blood pressure (mm/hg)	79.19 ± 9.68	77.70 ± 9.41	85.20 ± 8.56	−0.85	−1.3, −0.34
ACE-R: global (score)	86.82 ± 7.33	86.96 ± 7.56	86.20 ± 6.38	0.11	−0.38, 0.60
ACE-R: orientation (score)	16.89 ± 1.48	16.89 ± 1.54	16.90 ± 1.25	−0.01	−0.49, 0.48
ACE-R: memory (score)	20.48 ± 4.03	20.67 ± 3.83	19.70 ± 4.81	0.23	−0.26, 0.71
ACE-R: verbal fluency (score)	11.32 ± 2.02	11.35 ± 2.08	11.20 ± 1.79	0.08	−0.41, 0.56
ACE-R: comprehension (score)	23.31 ± 2.12	23.23 ± 2.15	23.65 ± 1.98	−0.21	−0.70, 0.28
ACE-R: visuospatial (score)	14.82 ± 1.41	14.83 ± 1.45	14.75 ± 1.25	0.06	−0.43, 0.55

^1^Data is reported for the overall sample and aggregated by sex as mean ± standard deviation (SD).

^2^The differences between males and females are reported as the standardized mean difference (SMD) with 95% confidence intervals (95% CI).

### 3.2 Heart rate variability and cognition

To examine the influence of cognitive function on cardiac autonomic dynamics, a structured rest–exercise–recovery protocol was implemented. This design enabled the characterization of non-linear trajectories in HRV indices as a function of cognitive performance, quantified using total scores from the Addenbrooke’s Cognitive Examination–Revised (ACE-R).

#### 3.2.1 At rest

Examination of the association between orientation scores and resting autonomic function indicated that higher orientation performance was moderately related to greater autonomic modulation, as reflected by SDNN (ρ = 0.331, *p* = 0.002). Comparable associations were observed for LF (ρ = 0.302, *p* = 0.005) and VLF (ρ = 0.236, *p* = 0.031). In contrast, elevated SNS index values (ρ = –0.240, *p* = 0.028) and higher Stress Index scores (ρ = –0.300, *p* = 0.006) were associated with poorer orientation performance.

#### 3.2.2 At exercise

Similarly, greater in-exercise HF power band (a marker of parasympathetic tone) was linked with better memory (ρ = 0.217, *p* = 0.048), orientation (ρ = 0.265, *p* = 0.015) and overall cognition scores (ρ = 0.228, *p* = 0.037).

#### 3.2.3 Dynamic HRV response conditioned by cognitive levels

Analysis of the multivariate hierarchical Bayesian regression models indicated a selective influence of cognitive function on autonomic responses. Cognitive performance affected only the trajectories of the LF and VLF components of HRV, as well as the composite parasympathetic index (PNS index).

**FIGURE 1:**
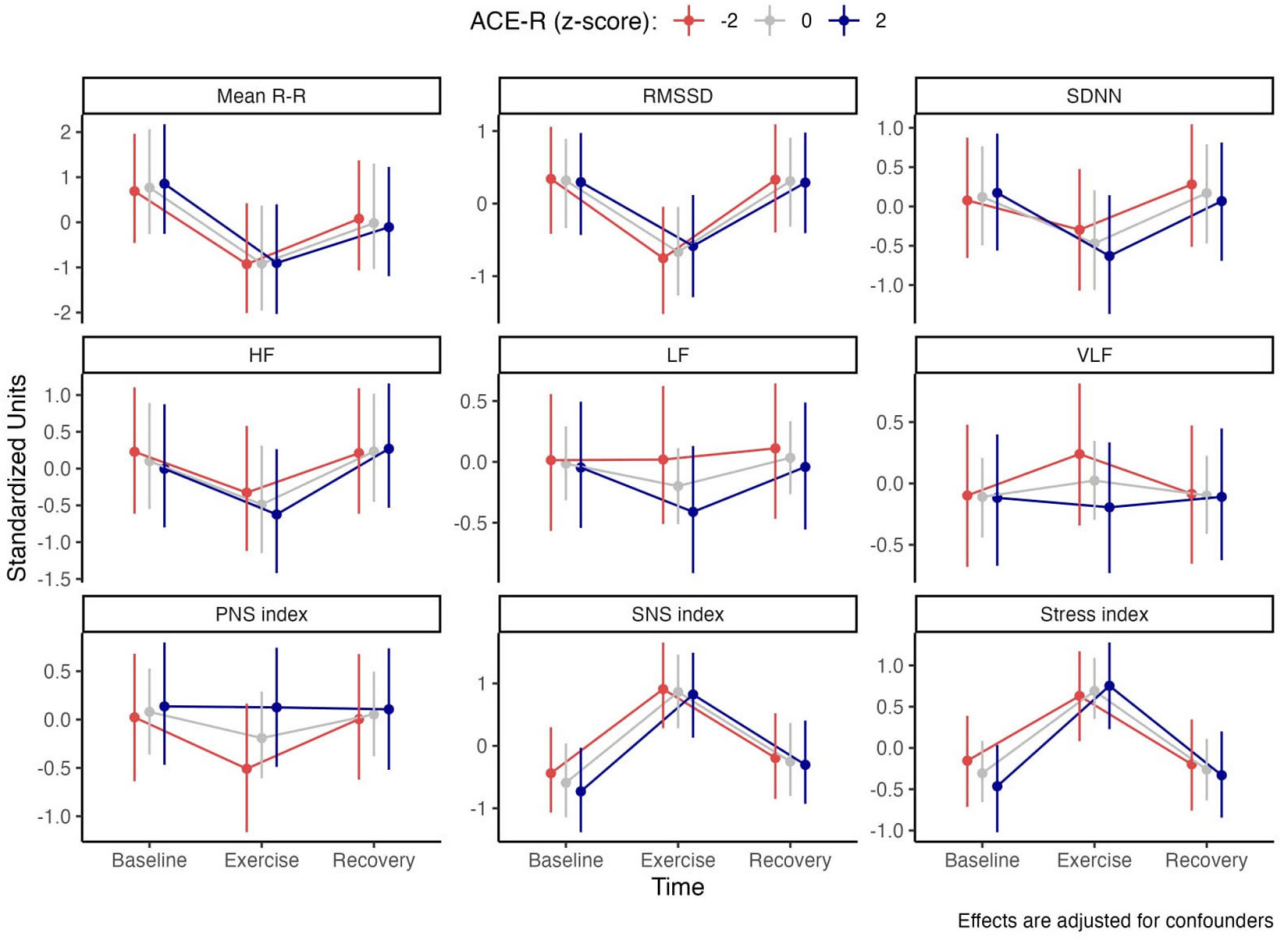
Predicted Exercise-induced cardiac autonomic trajectories. Model effects and 95% highest density interval (95% HDI) from a multivariate hierarchical Bayesian regression model illustrating the trajectories of HRV indices in response to the 2-min Step Test (TMST), capturing the onset and recovery dynamics, conditioned by different levels of cognition, denoted by different levels of ACE-R scores in standardized units. All effects are adjusted for confounding factors.

Higher ACE-R scores were associated with a more pronounced decline in LF and VLF values during exercise. LF is commonly interpreted as reflecting baroreflex sensitivity and sympathetic activity, whereas VLF is believed to capture slower physiological processes such as hormonal fluctuations, inflammatory activity, and circadian influences. Likewise, the PNS index, an indicator of parasympathetic modulation, showed a greater in-exercise reduction among individuals with lower ACE-R scores (Figure 1).

### 3.3 Confounding factors

Individuals with lower cardiac autonomic modulation, linked with lower parasympathetic and higher sympathetic indices at rest, tend to have greater BMI (RMSSD, ρ = −0.339, *p* = 0.002; SDNN, ρ = −0.289, *p* = 0.008; HF, ρ = −0.405, *p* < 0.001; PNS index, ρ = −0.292, *p* = 0.007; SNS index, ρ = 0.346, *p* = 0.001).

## 4 Discussion

This study examined the influence of cognitive ability on cardiac autonomic regulation in elderly individuals following a standardized protocol of rest, activity, and recovery. Bayesian hierarchical modeling and high-resolution HRV data demonstrate that cognitive status, evaluated through the Addenbrooke’s Cognitive Examination, exerts a selective and substantial influence on autonomic dynamics. The effect was most pronounced in the spectral HRV components (LF and VLF) and the composite PNS index, both at baseline and in response to the physiological stress induced by the TMST.

Cognitive performance did not uniformly affect all HRV parameters; rather, it appeared to influence specific autonomic characteristics linked to the CAN (6, 7). The findings suggest that reduced flexibility in cardiac autonomic modulation under mild physiological stress may act as an early neurophysiological marker associated with cognitive aging.

### 4.1 Cognitive performance and autonomic regulation at rest

Higher orientation and global cognition scores exhibited positive correlations with SDNN, LF, and VLF indices, while demonstrating negative correlations with sympathetic markers, including the SNS index and Baevsky’s Stress Index. This pattern indicates a more advantageous autonomic profile in individuals with superior cognitive status, marked by reduced sympathetic drive and increased overall variability, a configuration consistently associated with healthier aging trajectories (3, 35). The findings are consistent with prior research indicating that individuals with MCI or early Alzheimer’s disease generally demonstrate decreased HRV and reduced autonomic flexibility, presumably attributable to structural and functional changes in prefrontal and limbic regions critical to the CAN (4, 13).

The relationship between orientation and LF/VLF may indicate the overall integrity of cortical–subcortical communication, especially in circuits related to sensory integration, attention, and executive control (9). VLF power correlates with slow-acting physiological systems, such as hormonal, inflammatory, and metabolic processes. Consequently, reduced VLF in individuals with impaired cognition may suggest early dysregulation of these systems (10, 20). This interpretation aligns with evidence linking low-grade chronic inflammation and autonomic imbalance to accelerated cognitive decline and diminished neural resilience in aging (36, 37).

### 4.2 Cognitive performance modulates autonomic response during exercise

A significant finding of this study is the evidence that cognitive performance influences the autonomic response to the TMST. Higher cognitive scores correlated with increased HF power during exercise—signifying maintained parasympathetic activity under stress—and with more significant decreases in LF and VLF during the exertion phase (15, 17). In healthy adults, exercise usually causes the parasympathetic system to shut down and the sympathetic system to turn on. This is followed by a quick re-engagement of the vagus nerve during the early stages of recovery (38–40). People who have better cognitive function seem to have a more flexible autonomic response throughout these phases. This is becoming more and more seen as a sign of physiological and neural resilience in older adults (39).

Conversely, participants with diminished ACE-R scores demonstrated reduced autonomic transitions, as indicated by flatter LF and VLF trajectories. This pattern might mean that the baroreflex sensitivity is not working as well and that the body is less able to handle physiological stress (41, 42). These deficits are extensively documented in individuals with MCI and early Alzheimer’s disease, wherein disruptions in prefrontal-vagal pathways lead to reduced autonomic adaptability (9, 12, 13). The current findings augment the existing evidence by demonstrating that even a brief, submaximal functional challenge uncovers significant variations in dynamic autonomic regulation relative to cognitive status.

In general, these results show how useful autonomic markers could be as early signs of cognitive vulnerability. Dynamic responses to standardized physiological challenges may reveal subtle changes in autonomic function that are not apparent in static, resting HRV measures or baseline assessments.

### 4.3 Dynamic autonomic trajectories as early markers of cognitive vulnerability

The Bayesian multivariate framework facilitated the characterization of non-linear HRV trajectories during rest, exercise, and recovery. Cognitive performance selectively affected LF, VLF, and the PNS index, indicating that cognitive status interacts with particular autonomic regulatory mechanisms (5, 18).

People with higher cognitive scores had bigger drops in LF and VLF after exercise, which suggests that their baroreflex was more adaptable and their neurohormonal response to physical stress was better. This pattern aligns with recent research demonstrating that prolonged baroreflex sensitivity and efficient LF modulation are associated with improved cognitive performance and a reduced risk of long-term dementia (42). Conversely, individuals exhibiting diminished cognitive performance demonstrated flatter LF and VLF trajectories, accompanied by a more pronounced reduction in the PNS index. This indicates that their parasympathetic withdrawal was less efficacious, and their autonomic adaptability demonstrated diminished flexibility. Reduced autonomic responses have been noted in adults with mild cognitive impairment and early Alzheimer’s disease, supporting the hypothesis that initial disturbances in prefrontal–vagal pathways impede dynamic autonomic regulation (12). The results indicate that the autonomic system’s capacity for dynamic adaptation to a standardized physiological challenge may function as an early biomarker of cognitive vulnerability. This interpretation is consistent with the neurovisceral integration model, indicating that reduced autonomic flexibility signifies compromised prefrontal regulatory control.

### 4.4 Influence of BMI and cardiometabolic factors

A consistent pattern emerged, indicating that elevated BMI correlates with diminished autonomic function across various HRV indices, characterized by reduced RMSSD, SDNN, HF, and PNS index values, as well as increased SNS index values. This emphasizes the importance of BMI as a confounding variable in studies related to HRV and cognition. Excess adiposity is associated with autonomic dysregulation via insulin resistance, endothelial dysfunction, chronic low-grade inflammation, and diminished baroreflex sensitivity, all of which accelerate cognitive decline in older populations (5, 43). Older people may be more likely to get neurocardiovascular disease if they have a higher body mass index and less autonomic flexibility. Because metabolic stress can affect vagal tone and cognitive resilience on its own, the results show how important it is to include body composition and cardiometabolic factors in future studies of neurocardiac interactions.

### 4.5 Clinical relevance and implications for aging research

Combining a simple functional test like the TMST with HRV measurements is a cost-effective, scalable, and clinically useful way to check how well older adults’ autonomic systems are working. If longitudinal studies confirm this method, it might help find cognitive vulnerability or autonomic dysfunction early on. From a behavioral and functional perspective, autonomic flexibility during a brief standardized challenge may represent a physiological correlate of real-world adaptability in older adults, with potential links to mobility reserve, effort tolerance, and vulnerability to geriatric outcomes. Mechanistically, these dynamic responses are consistent with CAN-related regulation: cognitive status may modulate prefrontal–limbic control over vagal outflow and baroreflex-related oscillations, thereby shaping LF and VLF dynamics and the balance between parasympathetic withdrawal and recovery under mild stress. Given the well-established links between cognitive impairment and autonomic dysfunction, future studies should strengthen the validation of this approach by including older adults with diagnosed cognitive impairment or cognitive disability. Comparing dynamic HRV responses across clinically characterized cognitive strata (e.g., mild cognitive impairment and dementia) would clarify whether the observed HRV–cognition associations generalize across different levels of cognitive function and would enhance the clinical applicability of TMST-coupled autonomic assessment. Exercise-induced autonomic markers yield significant insights, as they indicate dynamic regulatory capacity during physiological stress and may demonstrate enhanced sensitivity relative to resting HRV alone (15, 17).

The results of this study demonstrate that cognitive performance affects autonomic dynamics, even under mild exertion, underscoring the importance of dynamic HRV assessments in geriatric evaluation. Resistance training, aerobic exercise, and mind–body practices like tai chi and yoga are examples of interventions that improve autonomic flexibility. These have been shown to improve vagal modulation and cognitive function (14, 43). HRV trajectories may serve as preliminary indicators of physiological dysregulation and as prospective biomarkers for evaluating the efficacy of targeted interventions designed to facilitate healthy aging.

### 4.6 Strengths and limitations

This study has several strong points, such as the use of a physiologically standardized protocol, strict Bayesian analytical methods, the Polar H10 sensor for high-quality HRV acquisition, and the ACE-III for validated cognitive assessment. The inclusion of two independent research centers enhances the ecological validity of the study and substantiates the notion that the methodology is applicable in diverse contexts. Importantly, the TMST-based protocol is low-cost and easily implementable, and the acquisition and analytical pipeline are described in a stepwise manner to facilitate reproducibility and independent replication across laboratories and countries. Nonetheless, replication in cohorts with different sociodemographic profiles, healthcare contexts, and medication patterns will be essential to confirm external validity.

Nonetheless, specific constraints must be acknowledged. The cross-sectional design limits causal inference, inhibiting determinations regarding whether autonomic alterations contribute to cognitive decline or arise as consequences thereof. The sample size was sufficient; however, the predominance of female participants, although indicative of regional demographic trends, may restrict broader generalizability. In addition, medication use is a relevant source of variability in older cohorts and may influence both resting and dynamic HRV. Common drug classes such as β-blockers, antihypertensives, and antidepressants can modify heart rate dynamics, baroreflex responsiveness, and sympathetic–parasympathetic balance, potentially attenuating or reshaping LF, HF, VLF patterns during exercise and recovery. Although our standardized protocol reduces situational noise, future studies should explicitly quantify medication exposure and incorporate it analytically (e.g., covariate adjustment, stratification by medication class, or sensitivity analyses excluding agents with strong autonomic effects) to further strengthen causal interpretation and clinical translation.

Future research would benefit from longitudinal designs, the integration of neuroimaging techniques to examine central autonomic pathways, and the evaluation of whether interventions that enhance autonomic flexibility can mitigate or postpone cognitive decline.

## 5 Conclusion

Older adults with better cognitive performance exhibit more favorable autonomic profiles and greater dynamic flexibility of cardiac autonomic regulation during a standardized exercise challenge. In contrast, individuals with lower cognitive scores show blunted autonomic transitions, suggesting early neurophysiological vulnerability. These findings support the concept that dynamic HRV responses to exercise may represent a sensitive, non-invasive marker of cognitive health and aging.

## Data Availability

The raw data supporting the conclusions of this article will be made available by the authors, without undue reservation.
